# Signalling pathways in the osteogenic differentiation of periodontal ligament stem cells

**DOI:** 10.1515/biol-2022-0706

**Published:** 2023-09-11

**Authors:** Liuyu Ru, Bowen Pan, Jiwei Zheng

**Affiliations:** School of Stomatology, Xuzhou Medical University, Xuzhou, Jiangsu, 221000, China; School of Medical Technology, Xuzhou Medical University, Xuzhou, Jiangsu, 221000, China; Department of Oral and Maxillofacial Surgery, Affiliated Hospital of Xuzhou Medical University, Xuzhou, Jiangsu, 221000, China

**Keywords:** inflammatory environment, periodontal ligament stem cells, osteogenic differentiation, signalling pathways

## Abstract

Periodontal ligament stem cells (PDLSCs) have multidirectional differentiation potential and self-renewal abilities and are important seed cells for the regenerative repair of periodontal tissues. In recent years, many studies have identified multiple signalling pathways involved in regulating the osteogenic differentiation of PDLSCs in an inflammatory environment. In this article, we review the osteogenic differentiation of PDLSCs in an inflammatory environment in terms of signalling pathways and provide new ideas for the regenerative treatment of periodontal tissues.

## Graphical Abstract

PDLSCs possess multidirectional differentiation potential and self-renewal ability. In the inflammatory environment, signalling pathways, such as Wnt/β-catenin, Wnt/Ca^2+^, Wnt/PCP, NF-κB, MAPK, BMPs, and PI3K/AKT, are involved in regulating the osteogenic differentiation of PDLSCs. In addition, multiple signalling pathways that interact with microRNAs play crucial roles in the osteogenic differentiation of PDLSCs.

**Figure:**
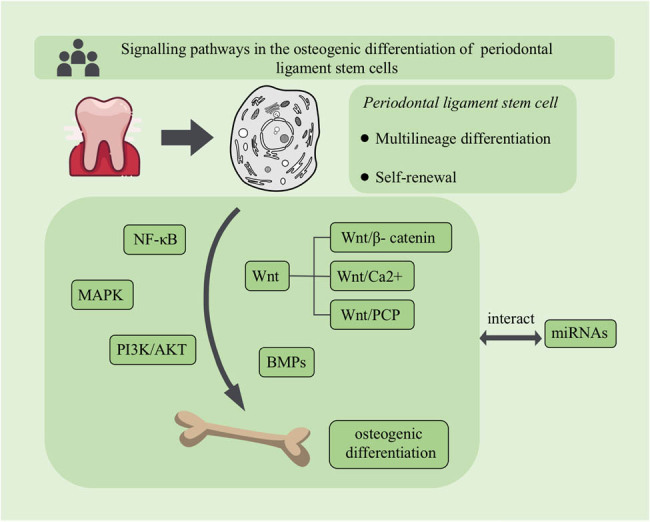

## 1 Preface

The oral cavity is the starting organ of the digestive tract and is connected to the external environment forwards by the fissure and backward by the pharynx via the isthmus. The oral cavity is an open organ where many microorganisms accumulate, and the oral inflammatory environment can be divided into two categories: pathological and nonpathological. The nonpathological oral inflammatory environment is a harmless inflammatory microenvironment maintained by the normal flora in the oral cavity through competition and mutual restraint between the host and the normal flora, and most microorganisms are normal flora and conditionally pathogenic bacteria, maintaining the balance of flora in the human body. When the balance between the flora in the oral cavity is disrupted due to a decrease in immunity, trauma, infection, allergy, or systemic disease, inflammatory diseases of the oral cavity will occur, resulting in the formation of a pathological inflammatory environment. There is a wide range of clinical oral inflammatory diseases, including oral ulceration, allergic stomatitis, chemo/radiotherapy-induced oral mucositis, periodontitis, necrotising inflammatory disease of the jaws, Sjogren syndrome (SS), and many more others. Of these, periodontitis is one of the most common clinical inflammatory diseases of the oral cavity and is by far one of the most dangerous to human oral health. Periodontitis is a chronic inflammation of the supporting tissues of the periodontium caused mainly by local factors, and oral bacteria are the initiating factors in its pathogenesis, while genetic and acquired factors of the host are the determining factors in the development and aggravation of periodontitis. If gingivitis is not treated in time, inflammation can spread from the gingiva to the periodontium, alveolar bone and dental bone and develop into periodontitis, eventually leading to the loosening and loss of teeth, resulting in masticatory dysfunction [1]. The goal of periodontal disease treatment is to eliminate bacterial plaque, control inflammation, and regenerate and rebuild periodontal tissue [2]. With the development of preventive dentistry, the concepts of regular oral check-ups, tartar removal, oral hygiene and plaque control have gradually become more popular. However, once periodontitis has occurred, the release of tumour necrosis factor (TNF), interleukins (ILs), and other inflammatory factors by inflammatory cells in response to external stimuli can mediate secondary tissue damage. If the development of this pathological inflammatory environment is not prevented in time, serious damage to periodontal tissues will result. Therefore, investigating how this pathological inflammatory environment damages periodontal tissues and the underlying mechanisms that prevent periodontal tissue regeneration may lead to new ways of treating periodontal disease and improving the prognosis of periodontal disease.

In recent years, with the rapid development of tissue engineering and stem cell technology, many studies have identified the use of mesenchymal stem cells (MSCs) as a promising therapeutic strategy for the treatment of oral inflammatory diseases [3]. In particular, the discovery of periodontal ligament stem cells (PDLSCs) has made it possible to regenerate periodontal tissues. PDLSCs have the potential to differentiate into a variety of mesenchymal cells, such as chondrocyte-like cells, adipocyte-like cells, and osteoblast-like cells. These are important seed cells for periodontal tissue regeneration and repair. Studies on the osteogenic differentiation of PDLSCs in inflammatory environments mostly involve *in vitro* experiments. Although there have been animal studies on the use of PDLSCs in periodontal tissue regeneration therapy, many of these animal models are not true models of pathological inflammation, and the cells have not been successfully implanted into the jawbone, making this research somewhat limited [4]. However, many studies have shown that the osteogenic differentiation of PDLSCs in an inflammatory environment is regulated by complex signalling pathways, and studying the mechanisms by which signalling pathways regulate the osteogenic differentiation of PDLSCs in a pathological oral inflammatory environment may be an important way to combat periodontal disease.

In this article, we review studies on the regulation of the osteogenic differentiation of PDLSCs by different signalling pathways in an inflammatory environment to provide inspiration to subsequent researchers.

## 2 Wnt signalling pathway

The Wnt signalling pathway is a complex network of protein interactions that mainly consists of the classical Wnt/β-catenin signalling pathway and the nonclassical Wnt/Ca^2+^ and Wnt/PCP signalling pathways [5].

### 2.1 Wnt/β-catenin signalling pathway

Previous studies have demonstrated that the Wnt/β-catenin signalling pathway plays an important role in activating the osteogenic differentiation potential of stem cells [6,7]. In the absence of Wnt signalling, glycogen synthase kinase-3β (GSK-3β) constitutively phosphorylates β-catenin and ubiquitously degrades it, thereby inhibiting β-catenin from entering the nucleus; in the presence of Wnt signalling, Wnt proteins bind with the FZD receptor (Frizzled) and low-density lipoprotein receptor-related protein 5/6 (LRP5/6) on the cell membrane. Then, a complex consisting of APC, Axin protein, casein kinase 1 (CK1), and glycogen synthase kinase 3 (GSK3) is recruited to the cell membrane through interactions with FZD, inhibiting GSK-3β- and CK-1α-induced phosphorylation of β-catenin. As a result, the amount of β-catenin in the cytoplasm increases and promotes β-catenin entry into the nucleus, which in turn activates the intranuclear lymphoid enhancer-binding factor-1 (LEF-1)/T lymphocyte factor (TCF) transcription system and participates in the regulation of cell cycle progression and differentiation [8].

It has been shown that the classical Wnt/β-catenin signalling pathway is involved in the osteogenic differentiation of PDLSCs, but it is still unclear whether the classical Wnt signalling pathway plays a positive or negative role in the osteogenic differentiation of PDLSCs. Many scholars found that the classical Wnt signalling pathway positively regulated the osteogenic differentiation of PDLSCs. Interestingly, the inflammatory microenvironment can inhibit the osteogenic differentiation of PDLSCs by activating the Wnt/β-catenin signalling pathway. Wu and Xi [9] found that activation of SIRT1 by resveratrol in an inflammatory microenvironment promoted osteogenic differentiation of PDLSCs and that this effect was partly associated with inhibition of the Wnt/β-catenin signalling pathway.

### 2.2 Wnt/Ca^2+^ signalling pathway

The binding of Wnt5a and Wnt11 to the FZD protein and LRP coreceptor on the cell membrane can activate PLC via G proteins, resulting in a transient increase in cytoplasmic Ca^2+^ concentrations and further activation of downstream CAMKII, CaN, and PKC [10]. Activated CaN can activate the cytoplasmic protein nuclear factor associated with T cells (NFAT) via dephosphorylation. Activated NFAT promotes the expression of several genes in neuronal, cardiac, and skeletal muscle cells, as well as proinflammatory genes in lymphocytes [11]. Activated CAMKII activates transcriptional growth factor β-activated kinase 1 (TAK-1) – NLK, which phosphorylates LEF/TCF, blocks the binding of the β-catenin-LEF/TCF complex to DNA, and inhibits its transcription.

Liu et al. [12] showed that the nonclassical Wnt/Ca^2+^ signalling pathway is involved in the osteogenic differentiation of PDLSCs in a chronic inflammatory microenvironment. After osteogenesis induction, CAMKII and NLK levels were significantly increased in H-PDLSCs and P-PDLSCs, but the levels were always lower in P-PDLSCs than in H-PDLSCs, indicating that the osteogenic differentiation of PDLSCs via the nonclassical Wnt/Ca^2+^ signalling pathway was somewhat inhibited in an inflammatory environment.

### 2.3 Wnt/PCP signalling pathway

In the atypical planar cell polarity pathway, Wnt is expressed upstream of PCP signalling and in a gradient, providing the polarity signal [13]. Wnt11 activates Dvl proteins in the cytoplasm by binding to Frizzled receptors on the cell membrane. On the one hand, Dvl proteins can activate Damm1, which dissociates into RhoA. Then, RhoA activates ROCK2, which in turn affects the formation of the cytoskeleton. On the other hand, Dvl proteins can activate Rac, which activates JNK and ultimately affects gene transcription [14].

The role of the Wnt/PCP signalling pathway in the development of cancer has been demonstrated previously [15]. Li et al. [16] found that miR-154-5p could regulate the osteogenic differentiation of adipose-derived mesenchymal stem cells via the Wnt/PCP signalling pathway in response to mechanical stress. No studies have reported the influence of the Wnt/PCP signalling pathway on the osteogenic differentiation of PDLSCs in an inflammatory environment.

### 2.4 Interaction between classical Wnt/β-catenin and nonclassical Wnt/Ca^2+^ signalling pathways in an inflammatory environment (Figure 1)

Current research suggests that classical and nonclassical Wnt signalling pathways play important roles in bone development and regeneration [17]. In a healthy state, a certain dynamic balance is maintained between classical and nonclassical Wnt signalling pathways; in an inflammatory environment, monocytes release the inflammatory factors TNF-α, IL-1β, and IL-6. The release of these inflammatory factors activates many signalling pathways and induces many diseases. Kong et al. [18] found that TNF-α was a key inflammatory factor. GSK3β phosphorylation and activation of the classical Wnt/β-catenin signalling pathway are key steps in the inhibition of the osteogenic differentiation of PDLSCs by TNF-α. Liu et al. [19] found that inhibition of β-catenin protein in the classical signalling pathway during osteogenic induction enhanced the expression level of NLK, a core protein of the nonclassical Wnt signalling pathway, and enhanced the osteogenic differentiation of P-PDLSCs. This finding suggests that enhancing the classical Wnt signalling pathway and weakening the nonclassical Wnt signalling pathway in the inflammatory microenvironment may be potential mechanisms leading to enhanced proliferation and reduced osteogenic differentiation in P-PDLSCs. Targeted inhibition of the β-catenin protein, which is activated by chronic periodontitis, in the classical signalling pathway and maintaining the balance between the classical and nonclassical Wnt signalling pathways may improve the level of osteogenic differentiation of P-PDLSCs and provide new strategies for the regenerative treatment of periodontal tissues.

**Figure 1:**
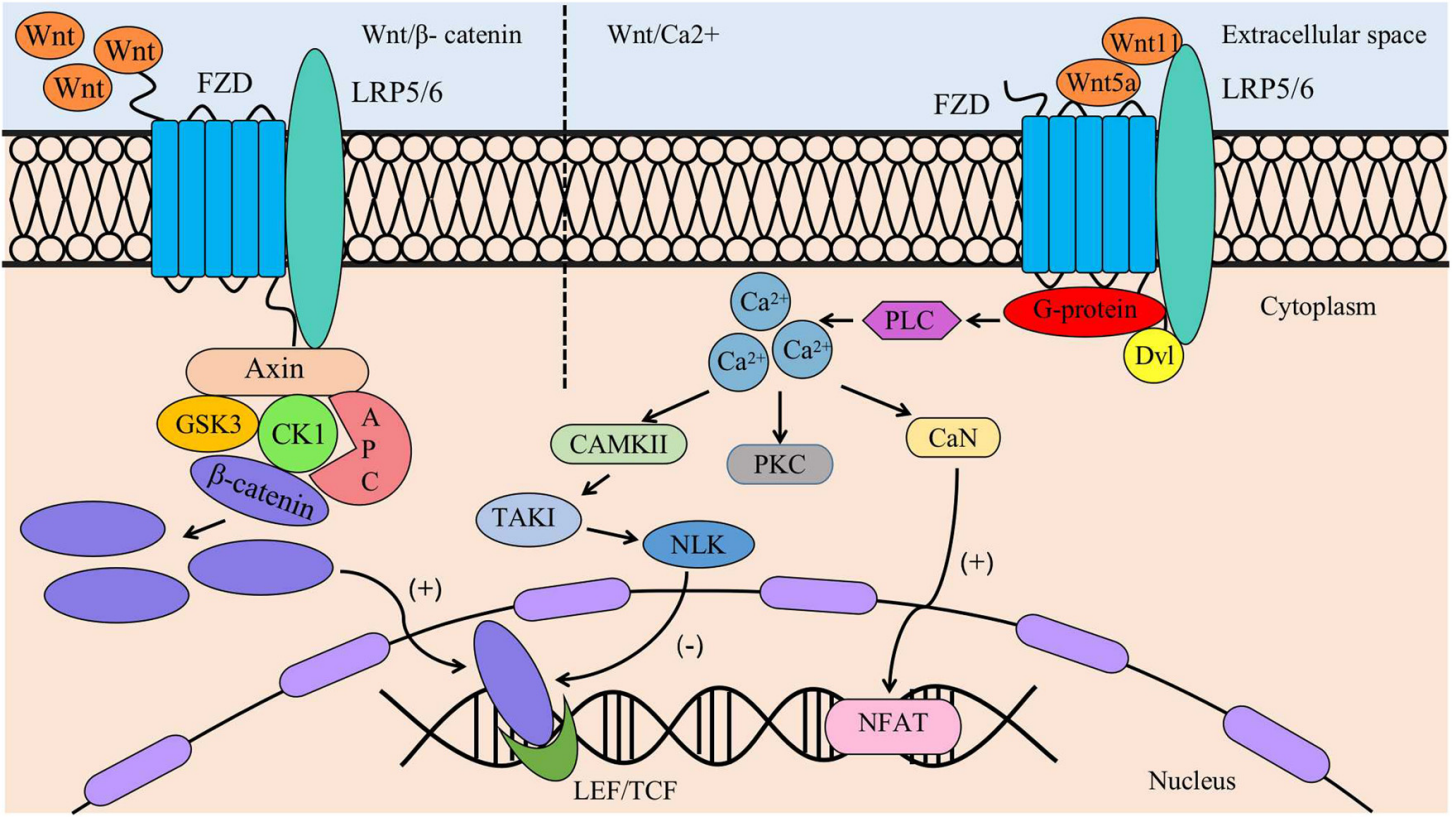
Interaction of Wnt/β-catenin and Wnt/Ca^2+^ signalling pathways in an inflammatory environment. In the Wnt/β-catenin signalling pathway, Wnt signals are recognised and bound by FZD receptors and LRP5/6 receptors on the cell membrane. A complex consisting of APC, Axin, CK1, and GSK3 is recruited to the cell membrane through interaction with FZD. This complex facilitates an increase in β-catenin levels and promotes the entry of β-catenin into the nucleus, where it activates LEF/TCF. In the Wnt/Ca^2+^ signalling pathway, Wnt5a and Wnt11 bind to the FZD and LRP co-receptors on the cell membrane. This binding activates PLC through G proteins, resulting in a transient increase in cytoplasmic Ca^2+^ concentration. This increase further triggers downstream signalling involving CAMKII, CaN, and PKC. Activated CAMKII can then activate TAKI-NLK, and NLK phosphorylates LEF/TCF, thereby preventing the binding of the β-catenin-LEF/TCF complex to DNA.

The Wnt signalling pathway has been relatively well studied, but the complex regulatory mechanisms through which inflammatory factors activate the Wnt signalling pathway in an inflammatory environment remain unclear. Furthermore, the underlying mechanisms of the interactions among Wnt signalling pathways remain obscure. Investigating the activation mechanisms of Wnt signalling pathways and the interaction mechanisms between classical and nonclassical Wnt signalling pathways to identify key targets to block the development of chronic periodontitis could be invaluable in the clinical management of periodontal disease.

## 3 NF-κB signalling pathway (Figure 2)

Two NF-κB activation pathways exist in cells. The classical pathway is mainly activated by TNF, IL-1, and Toll-like receptor 4 (TLR4), is mainly dependent on IKKβ and NEMO, and leads to IκBα phosphorylation and p65-containing heterodimeric nuclear translocation. The nonclassical pathway is induced by specific members of the TNF cytokine family and relies primarily on IKKα-mediated phosphorylation of p100 associated with RelB, leading to partial processing of p100 and production of the p52-RelB complex [20]. Numerous studies have confirmed the role of the NF-κB signalling pathway in regulating the osteogenic differentiation of PDLSCs. Cell signalling molecules act mainly through classical pathways to activate surface receptors on the cell membrane and are involved in regulating the osteogenic differentiation of PDLSCs. The inflammatory factor TNF-α forms a complex by binding to PDLSC surface receptors, and TRAF activates TAK1 and TAB via ubiquitination, which phosphorylate the downstream IκB kinase (IKK) and further phosphorylate IκB. Then, IκB phosphorylation is followed by ubiquitinated degradation in the cytoplasm, and the p50/p65 complex is released and transferred to the nucleus, where it binds to DNA to induce transcription. This reduces the expression of osteogenic markers such as osteopontin (OPN), osteocalcin (OCN), zinc finger family transcription factors (Osx), and Runx2, leading to a reduction in the osteogenic differentiation of PDLSCs [21].

**Figure 2:**
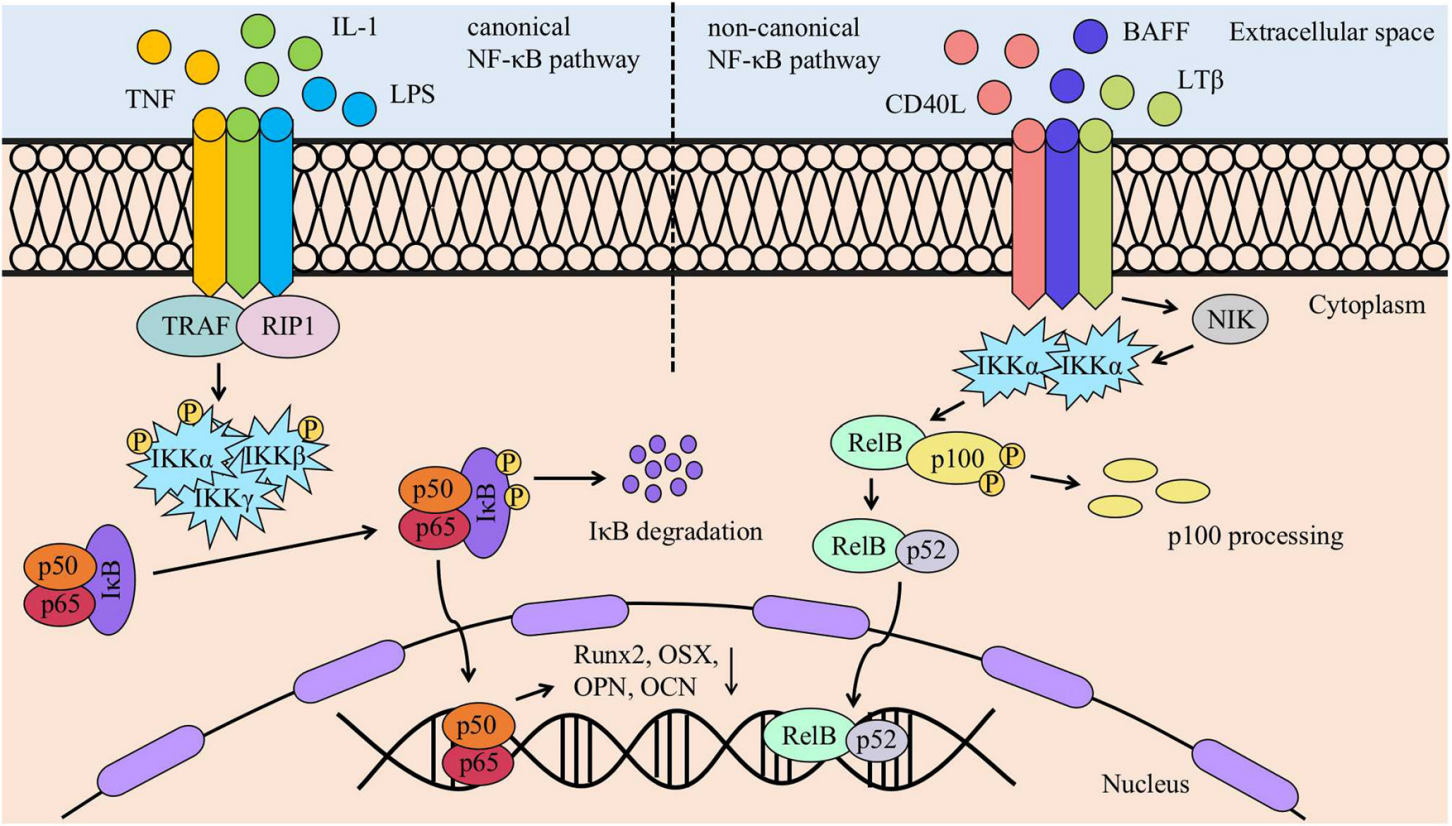
NF-κB signalling pathway. In the canonical NF-κB signalling pathway, inflammatory factors such as TNF, IL-1, and LPS bind to cell surface receptors, phosphorylating downstream IκB kinase (IKK) and subsequently phosphorylating IκB. Phosphorylated IκB is degraded in the cytoplasm, releasing the p50/p65 complex. This complex translocates to the nucleus and binds to DNA, resulting in the downregulation of osteogenesis-related markers, such as OPN, OCN, Osx, and Runx2. In the non-canonical NF-κB signalling pathway, cytokines such as CD40L, BAFF, and LTβ bind to cell surface receptors, promoting the activation of NIK and IKKα. IKKα phosphorylates p100, leading to partial processing of p100 and the formation of the p52–RelB complex. Subsequently, the p52–RelB complex enters the nucleus and binds to DNA.

In chronic periodontitis, there is a mutually reinforcing relationship between the NF-κB signalling pathway and the inflammatory environment. Inflammatory factors such as TNF-α and IL-1β in the inflammatory environment activate the NF-κB signalling pathway, while the NF-κB signalling pathway promotes the expression of these inflammation-related factors, creating a vicious cycle that exacerbates the development of chronic periodontitis [22]. Li and Zhou [23] found that the inflammatory factor IL-1β could activate the NF-κB signalling pathway, leading to a decrease in the osteogenic differentiation of PDLSCs and that inhibiting the NF-κB signalling pathway improved the osteogenic differentiation of PDLSCs. Zhang et al. [24] found that knockdown of Src homology-2 domain containing protein tyrosine phosphatase-2 (SHP2) could promote the osteogenic differentiation of PDLSCs by inhibiting the MAPK/NF-κB signalling pathway under inflammatory conditions. Paradoxically, knockdown of SHP2 can activate the NF-κB signalling pathway and reduce the osteogenic differentiation of PDLSCs under normal conditions. No studies have yet determined the reasons why knockdown of SHP2 in different environments has completely opposite effects on the osteogenic differentiation capacity of PDLSCs.

The main pathogenic factor in periodontitis is bacteria, and lipopolysaccharide (LPS) is a cell wall component of gram-negative bacteria. Many studies have shown the role of LPS in the pathogenesis of periodontitis. Dong and Shuang [25] showed that high concentrations of LPS (10 μg/mL) inhibited the osteogenic differentiation of PDLSCs, whereas low concentrations of LPS (0.1 μg/mL) promoted the osteogenic differentiation of PDLSCs. However, uncontrolled periodontitis eventually creates an environment with high concentrations of LPS, which inhibits the osteogenic differentiation of PDLSCs. LPS is mainly recognised by host Toll-like receptor 4 (TLR4) and stimulates the NF-κB signalling pathway, thereby reducing the osteogenic differentiation capacity of human PDLSCs. Interestingly, the osteogenic differentiation capacity of bone marrow mesenchymal stromal cells was not affected. Blocking the TLR4 or NF-κB signalling pathway could improve the osteogenic differentiation of PDLSCs, providing a new strategy for periodontal regeneration therapy [26]. Wang et al. [27] showed that LPS stimulation significantly promoted the expression of EZH2 and H3K27me3 in PDLSCs and that knockdown of EZH2 could promote osteogenic differentiation of PDLSCs by inhibiting the NF-κB signalling pathway. Furthermore, knockdown of EZH2 can prevent the LPS-induced upregulation of *TNFα*, *IL1β*, and IL6 as well as the inhibition of proliferation and osteogenic differentiation of PDLSCs, and the EZH2-TLR4/MyD88/NF-κB axis may be a new target in periodontal tissue regeneration therapy.

It has long been known that the NF-κB signalling pathway plays a negative role in the osteogenic differentiation of PDLSCs and that activation of the NF-κB signalling pathway in an inflammatory environment is one of the key factors that reduces the regenerative capacity of periodontal tissues. Many studies have focused on targeting the NF-κB signalling pathway to improve periodontitis-induced alveolar bone loss and enhance the osteogenic differentiation of stem cells. For example, reducing the expression of some proteins upstream or downstream of the NF-κB signalling pathway and blocking activation of the NF-κB signalling pathway have provided many new targets for periodontal tissue regeneration therapy. However, many studies are still in the *in vitro* stage, and more research is needed to confirm the safety and feasibility of this strategy in the treatment of periodontitis. Most of the drugs currently used in the treatment of inflammatory diseases, such as NSAIDs, anti-rheumatic drugs, cyclosporine A, and corticosteroids, have inhibitory effects on NF-κB activity [28], providing us with new ideas for the treatment of periodontitis.

## 4 MAPK signalling pathway (Figure 3)

Mitogen-activated protein kinases (MAPKs) are a group of serine-threonine protein kinases that can be activated by different extracellular stimuli. All eukaryotic cells express MAPKs, which consist of a three-tier signalling pathway: MAPK, MAP kinase kinase, and MAP kinase kinase of MAPK kinase. These three levels of signalling are activated sequentially and are involved in the regulation of cell proliferation, differentiation, stress, inflammation, apoptosis, and carcinogenesis. The MAPK family consists of four main subfamilies: extracellular signal-regulated protein kinase 1/2 (ERK1/2), p38 MAPK, c-Jun amino-terminal kinase (JNK), and ERK5. Cascade activation of MAPK is the intersection of many signalling pathways; therefore, MAPK is inextricably linked to the development of many diseases. 

**Figure 3:**
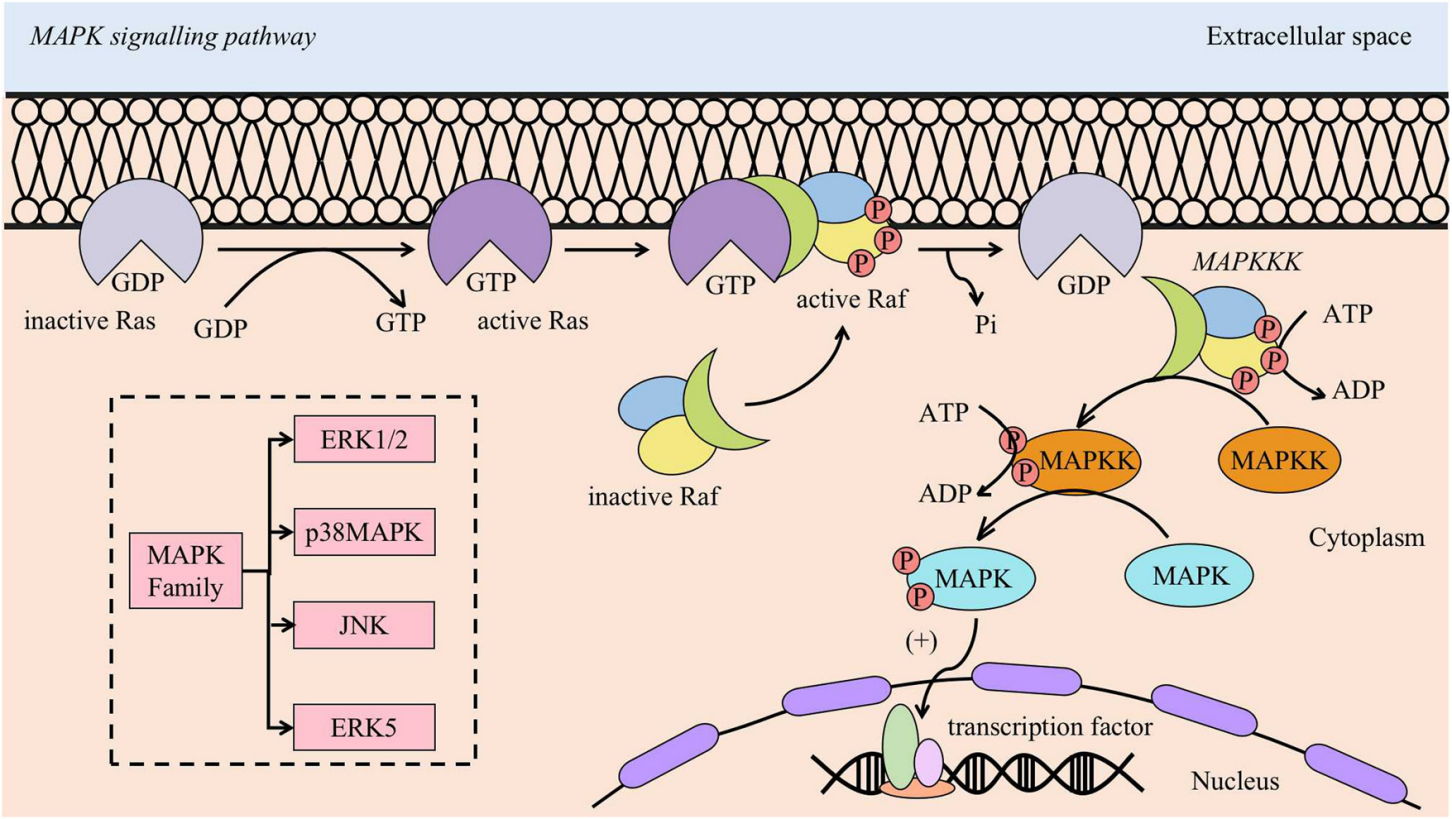
MAPK signalling pathway. The activation of the MAPK signalling pathway begins with the conversion of GDP to GTP, which activates Ras. Activated Ras then recognises, binds to, and activates Raf. Subsequently, hydrolysis of GTP leads to the separation of active Raf from Ras. The active Raf, known as MAPKKK, further activates MAPKK. Finally, MAPKK activates MAPK. The active MAPK molecule translocates to the nucleus and triggers the activation of various transcription factors.

A number of studies have shown that the ERK1/2 and p38 MAPK subfamilies are involved in the expression of osteogenic genes and bone formation. Kim et al. [29] demonstrated that there are potential regulators in the ERK/MAPK signalling pathway associated with osteogenic differentiation, suggesting that the ERK/MAPK pathway is involved in regulating osteogenic differentiation *in vivo*. Yong et al. [30] showed the interaction between the MAPK and β-catenin signalling pathways during orthodontic tooth movement (OTM), suggesting the potential of MAPK signalling pathways to regulate osteogenic differentiation in PDLSCs.

Although there have been many studies on the role of the MAPK family in osteogenic differentiation *in vivo*, there have been fewer studies of MAPK pathway involvement in regulating osteogenic differentiation in the inflammatory setting. The p38MAPK subfamily pathway has been shown to be involved in the osteogenic differentiation of stem cells derived from inflammatory tissues. Nie et al. [31] demonstrated that the p38MAPK signalling pathway regulates the osteogenic differentiation of P-PDLSCs. In an inflammatory setting, the levels of total p38 increase, but the relative levels of phosphorylated p38 (p-p38), which is involved in osteogenic differentiation, are reduced, suggesting that p38MAPK is involved in the cellular inflammatory response and that the P38MAPK signalling pathway may be a key factor in the reduction in the osteogenic differentiation of PDLSCs in periodontitis.

Given the important role of the MAPK signalling pathway in the osteogenic differentiation of stem cells, there have been many studies targeting the MAPK pathway to improve the osteogenic differentiation potential of PDLSCs in an inflammatory environment. Wang et al. [32] showed that erythropoietin (EPO) promoted the osteogenic differentiation of H-PDLSCs and P-PDLSCs through the p38MAPK signalling pathway. However, studies on the potential of EPO to improve the osteogenic differentiation of P-PDLSCs are still at the *in vitro* stage, and there is a lack of animal studies to confirm its role in promoting the osteogenic differentiation of P-PDLSCs *in vivo*. Yan et al. [33] found that cannabinoid receptor I (CB1) could promote the osteogenic differentiation of PDLSCs through the JNK and p38MAPK signalling pathways in an inflammatory environment, which could be useful for the treatment of periodontitis.

A search of major databases revealed that research on MAPK signalling pathway regulation of the osteogenic differentiation of PDLSCs in inflammatory states is still in its infancy. However, a recent study used the MAPK signalling pathway as a key target, especially the p38MAPK pathway, which is closely related to inflammation [34], and by regulating its upstream or downstream gene expression, as well as the signalling pathway that interacts with the MAPK pathway, it is expected to provide new targets for further development of anti-inflammatory drugs and new ideas for the clinical treatment of periodontitis diseases.

## 5 BMP signalling pathway (Figure 4)

Bone morphogenetic protein (BMP) signalling molecules belong to the transforming growth factor-β (TGF-β) protein superfamily, which plays an important role in the formation of bone and cartilage. Previous studies have identified BMP3 as a negative regulator of bone density and BMP13 as a strong inhibitor of bone formation. Moreover, BMP2, 4, 6, 7, and 9 have potent osteoinductive activity and play important roles in maintaining bone homeostasis in humans [35]. 

**Figure 4:**
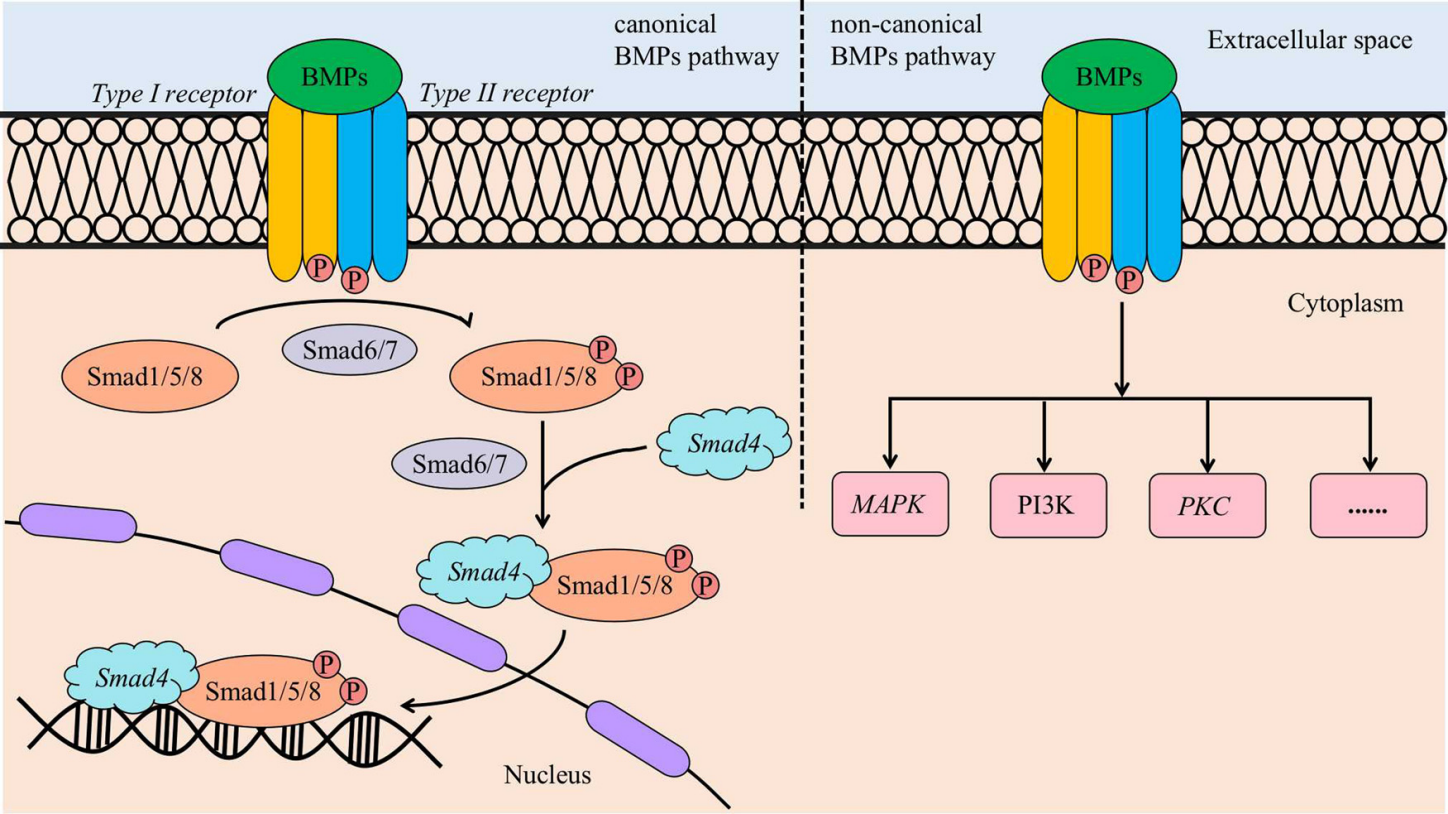
BMP signalling pathway. In the canonical BMP signalling pathway, BMP signalling molecules bind and activate the type I receptor and type II receptor. This binding leads to the phosphorylation of Smad1/5/8, with the involvement of Smad6/7. Phosphorylated Smad1/5/8 then forms a complex with Smad4 and translocates to the nucleus, where it regulates gene expression. In the non-canonical BMP signalling pathway, BMP signalling molecules can regulate gene expression by activating various signalling pathways, such as MAPK, PI3K, and PKC.

A study showed that the BMP signalling pathway could activate the MAPK pathway to participate in the osteogenic differentiation of stem cells. Li et al. [36] showed that the expression of BMP2, BMP7, and BMP9 could mediate the osteogenic differentiation of immortalised odontoblasts. Lei et al. [37] found that adenoviral vector-mediated bone morphogenetic protein 9 (Ad-BMP9) had a strong ability to promote the osteogenic differentiation of PDLSCs and that BMP9 promoted the osteogenic differentiation of PDLSCs via the BMP-ERK5 signalling pathway in an inflammatory environment. Wang et al. [38] found that NEL-like protein 1 (NELL1) could downregulate the expression of inflammatory genes in periodontitis and promote the BMP9-induced osteogenic differentiation of P-PDLSCs, which is a process in which the MAPK/p38/ERK pathway is also involved. In addition, the same study suggested that the development of NELL1- and BMP9-related drugs would be promising for the treatment of periodontitis-induced alveolar bone loss.

Many studies have shown that the BMP/Smad pathway plays an important role in the osteogenic differentiation of stem cells. After BMP binds to receptors on the corresponding cell membrane, it can phosphorylate Smad and activate the BMP signalling pathway, thereby regulating the expression of osteogenic-related markers and promoting the osteogenic differentiation of stem cells. Wang et al. [39] found that SHED-Exos could promote the osteogenic differentiation of PDLSCs in periodontitis, which may be related to the activation of the BMP/Smad and Wnt/β-catenin signalling pathways. According to Wang et al. [40], low-level laser irradiation promotes the proliferation and osteogenic differentiation of PDLSCs through the BMP/Smad signalling pathway. Gao et al. [41] found that connective tissue growth factor (CTGF) could enhance BMP2-induced osteogenic differentiation of PDLSCs, which might be related to the interaction between the Smad pathway and CTGF.

Many studies have demonstrated the role of the BMP signalling pathway in the development of periodontal disease. The search for targets related to the BMP signalling pathway may provide new ideas for the treatment of periodontal diseases. Liu et al. [42] showed that knocking down miR-155 in an inflammatory environment increased the level of osteogenic markers and increased the number of calcium nodules produced, suggesting that knocking down miR-155 could promote the osteogenic differentiation of P-PDLSCs, and mechanism was related to BMP5 and BMP10. The role of the BMP signalling pathway in the osteogenic differentiation of P-PDLSCs has been less well investigated, and further studies on the mechanisms and key targets by which the BMP signalling pathway regulates the osteogenic differentiation of PDLSCs are expected to provide new clinical treatment ideas for periodontal diseases.

## 6 PI3K/AKT signalling pathway

PI3K is an intracellular phosphatidylinositol kinase that consists of the regulatory subunit p85 and the catalytic subunit p110. PI3K can be divided into three classes, and the most widely studied is class I PI3K. When PI3K binds to the corresponding receptor on the cell membrane, the receptor activates the regulatory subunit p85 and recruits the catalytic subunit p110, which in turn catalyses the production of PI3P from PIP2 on the inner surface of the membrane. PI3P acts as a second messenger that can further activate AKT, which is an important signalling molecule downstream of PI3K that has at least three forms: AKT1, AKT2, and AKT3. Activated AKT plays an important physiological role in regulating cell proliferation, differentiation, development, and apoptosis by phosphorylating various downstream signalling molecules.

Mammalian target of rapamycin (mTOR) is an important signalling molecule downstream of the PI3K/AKT signalling pathway. The PI3K/AKT/mTOR signalling pathway is involved in regulating the osteogenic differentiation of PDLSCs [43]. Zhao et al. [44] previously found that rutin promoted the osteogenic differentiation of PDLSCs through the PI3K/AKT signalling pathway in an LPS-induced inflammatory environment. Zhao et al. [45] found that rutin could protect PDLSCs from TNF-α-induced decreases in osteogenic differentiation by inhibiting mTOR phosphorylation. Furthermore, it has been shown that transforming growth factor β1 (TGF-β1) can affect osteoblast activity *in vivo* through the PI3K/AKT/mTOR/S6K1 signalling pathway [46], but the effect of TGF-β1 on the osteogenic differentiation of PDLSCs, as well as other odontogenic stem cells, has not been reported.

In addition to inducing the osteogenic differentiation of PDLSCs through the mTOR pathway, a study by Xiong [47] showed that suitable concentrations of curcumin could promote the osteogenic differentiation of PDLSCs through the PI3K/AKT/Nrf2 signalling pathway. Curcumin is an agonist of nuclear factor erythroid-2-related factor 2 (Nrf2), which has various pharmacological effects, such as antitumour, antibacterial, anti-inflammatory, antioxidant, and immunomodulatory effects, and many studies have confirmed the role of curcumin in the treatment of periodontitis [48]. Nrf2 is a transcription factor associated with oxidative stress and inflammation, and Nrf2 activation enhances cellular resistance to chemical carcinogens and inflammation [49]. However, little research has been done on its role in osteogenic differentiation.

**Figure 5:**
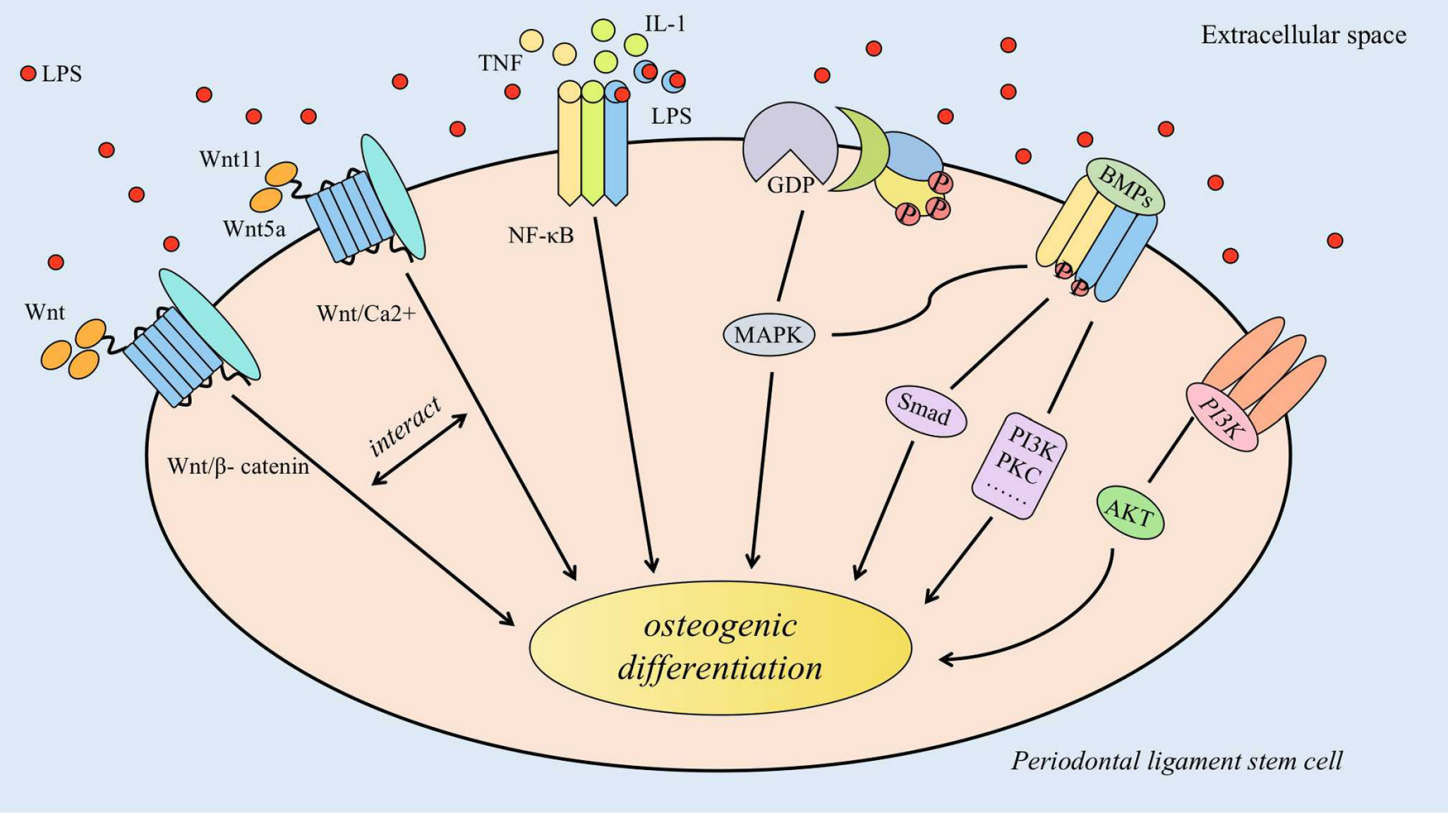
Summary of signalling pathways. In the inflammatory environment, multiple signalling pathways play crucial roles in regulating the osteogenic differentiation of PDLSCs. These signalling pathways include Wnt/β-catenin, Wnt/Ca^2+^, NF-κB, MAPK, BMPs, PI3K/AKT, and others. The interaction between the Wnt/β-catenin signalling pathway and the Wnt/Ca^2+^ signalling pathway also influences the osteogenic differentiation of PDLSCs. Moreover, the BMP signalling pathway is involved in regulating the osteogenic differentiation of PDLSCs through various signalling pathways, such as the canonical Smad pathway as well as non-canonical pathways like MAPK, PI3K, and PKC.

Although many studies have confirmed the role of the PI3K/AKT signalling pathway in osteogenic differentiation, there have been few studies on the osteogenic differentiation of PDLSCs by the PI3K/AKT signalling pathway in an inflammatory environment. Further studies on the complex mechanisms by which the PI3K/AKT pathway regulates the osteogenic differentiation of P-PDLSCs could identify new targets for the treatment of periodontitis (Figure 5).

## 7 Interaction between signalling pathways and microRNAs (miRNA)

miRNAs are a class of noncoding single-stranded RNA molecules, approximately 19–24 nt in length that are encoded by endogenous genes. miRNAs bind to the 3′UTR or coding region of target mRNA sequences through complementary base pairing, thereby directing the silencing complex (RISC) to degrade the RNA or prevent the translation of mRNA into protein.

Many studies have shown an inextricable relationship between miRNAs and signalling pathways, and the regulation of cellular signalling pathways via miRNAs is expected to provide new approaches for the treatment of some diseases. Zhong et al. [50] found that the inflammatory factor IL-1β activated the transcription of miR-31 through p38 and JNK MAP kinase and its downstream transcription factors GATA2, c-Fos, and c-Jun, which negatively regulated the expression of E-selectin, thereby inhibiting the adhesion and migration of colon cancer cells to the endothelium and reducing the metastatic potential of colon cancer cells. Later, Zhong et al. [51] showed that activation of p38 could induce the production of miR-146a and miR-181b, thereby inhibiting NF-κB-mediated E-selectin expression and the E-selectin-dependent metastatic ability of colon cancer cells. Moreover, p38 could increase E-selectin expression by reducing miR-31 at the posttranscriptional level. Moreover, a review by Zhong et al. [52] showed that endothelial miRNAs played an important role in many inflammation-related diseases by regulating the NK-κB signalling pathway and inhibiting the expression of cell adhesion molecules. Periodontitis is a classic inflammation-related disease, suggesting that we may be able to target miRNA transcriptional regulation as a new approach to treat periodontitis.

In recent years, many scholars have found that miRNAs can regulate the osteogenic differentiation of PDLSCs through signalling pathways. Chen and Wu [53] found that miRNA-132 overexpression could promote the osteogenic differentiation of PDLSCs through the Wnt/β-catenin signalling pathway. Moreover, Hu et al. [54] found that miRNA-222 overexpression could reduce the expression of Smad2 and Smad7 and thus inhibit the osteogenic differentiation of PDLSCs. These studies suggest that the interaction between miRNAs and signalling pathways may have important effects on the osteogenic differentiation of PDLSCs, further providing new ideas for regulating the osteogenic differentiation of PDLSCs.

## 8 Summary and outlook

In this review, the effects of different signalling pathways on the osteogenic differentiation of PDLSCs in the context of periodontitis were summarised through a search of national and international databases. The current study showed that Wnt, NF-κB, MAPK, BMP, PI3K/AKT, and other signalling pathways play important roles in the osteogenic differentiation of P-PDLSCs. These signalling pathways have independent transduction mechanisms and undergo crosstalk, and together, these pathways govern the osteogenic differentiation of P-PDLSCs. The aim of this article was to elucidate the mechanisms through which these signalling pathways regulate the osteogenic differentiation of P-PDLSCs and to show that the Wnt and NF-κB signalling pathways have been studied more frequently and that these two signalling pathways play major roles in osteogenic differentiation, while the MAPK, BMP, and PI3K/AKT signalling pathways have been less studied and need to be further explored.

Controlling the progression of inflammation is currently the main strategy in the treatment of periodontitis. Different anti-inflammatory drugs have different anti-inflammatory mechanisms, and many studies have shown that anti-inflammatory drugs exert their effects through signalling pathways, which raises many questions about drug use options. The antagonistic or synergistic effects of several anti-inflammatory drugs when used together can affect the efficacy of these drugs. The nature of the inflammatory response is defensive, and the misuse of anti-inflammatory drugs can be counterproductive. As a result, studying different signalling pathways in the pathological oral inflammatory environment may provide new insights into drug use.

Furthermore, anti-inflammatory treatment can only stop the further development of inflammation but cannot compensate for the tissue damage already caused by inflammation. Therefore, repairing periodontal tissue, inducing the regeneration of bone and alveolar bone and re-establishing a normal occlusal relationship are important strategies to improve the prognosis of periodontitis and to improve the quality of life of patients with periodontitis. With the development of bioinformatics and tissue engineering technologies, the interaction network between different signalling pathways can be constructed through bioinformatics analysis, and key targets for osteogenic differentiation can be identified. Through the extraction and combination of natural compounds acting on the targets, it is expected that periodontal tissue regeneration inducers with good efficacy can be developed, providing new ideas to improve the prognosis of periodontitis.
